# RETRACTION: Clinicopathological Significance of the MicroRNA‐146a/WASP‐Family Verprolin‐Homologous Protein‐2 Axis in Gastric Cancer

**DOI:** 10.1111/cas.70327

**Published:** 2026-01-19

## Abstract

**RETRACTION**: YaoQ. , TuC. , LuD. , ZouY. , LiuH. , and ZhangS. , “Clinicopathological Significance of the MicroRNA‐146a/WASP‐Family Verprolin‐Homologous Protein‐2 Axis in Gastric Cancer,” Cancer Science 108, no. 7 (2017): 1285–1292, 10.1111/cas.13254.28387985 PMC5497796

The above article, published online on 07 April 2017 in Wiley Online Library (wileyonlinelibrary.com), has been retracted by agreement between the journal Editor‐in‐Chief, Masanori Hatakeyama; the Japanese Cancer Association; and John Wiley & Sons Australia, Ltd. The retraction has been agreed upon following concerns about the reliability of data and images raised by a third party. An investigation also identified duplication of images between Figures 2b and 2c. The authors did not respond to invitations to comment on these concerns nor provide supporting data to support the findings. The editors therefore consider the results and conclusions of this article unreliable. The authors did not respond to our notice of retraction.

**RETRACTION**: Q. Yao , C. Tu , D. Lu , Y. Zou , H. Liu , and S. Zhang , “Clinicopathological Significance of the MicroRNA‐146a/WASP‐Family Verprolin‐Homologous Protein‐2 Axis in Gastric Cancer,” Cancer Science 108, no. 7 (2017): 1285–1292, 10.1111/cas.13254.28387985 PMC5497796

The above article, published online on 07 April 2017 in Wiley Online Library (wileyonlinelibrary.com), has been retracted by agreement between the journal Editor‐in‐Chief, Masanori Hatakeyama; the Japanese Cancer Association; and John Wiley & Sons Australia, Ltd. The retraction has been agreed upon following concerns about the reliability of data and images raised by a third party. An investigation also identified duplication of images between Figures 2b and 2c. The authors did not respond to invitations to comment on these concerns nor provide supporting data to support the findings. The editors therefore consider the results and conclusions of this article unreliable. The authors did not respond to our notice of retraction.

